# Evaluating the Performance of Speaker Recognition Solutions in E-Commerce Applications

**DOI:** 10.3390/s21186231

**Published:** 2021-09-17

**Authors:** Olja Krčadinac, Uroš Šošević, Dušan Starčević

**Affiliations:** Department for Information Technology, Faculty of Organizational Sciences, University of Belgrade, 11000 Belgrade, Serbia; uros.sosevic@fon.bg.ac.rs (U.Š.); dusan.starcevic@fon.bg.ac.rs (D.S.)

**Keywords:** speaker recognition, biometrics, e-commerce applications, identity management systems

## Abstract

Two important tasks in many e-commerce applications are identity verification of the user accessing the system and determining the level of rights that the user has for accessing and manipulating system’s resources. The performance of these tasks is directly dependent on the certainty of establishing the identity of the user. The main research focus of this paper is user identity verification approach based on voice recognition techniques. The paper presents research results connected to the usage of open-source speaker recognition technologies in e-commerce applications with an emphasis on evaluating the performance of the algorithms they use. Four open-source speaker recognition solutions (SPEAR, MARF, ALIZE, and HTK) have been evaluated in cases of mismatched conditions during training and recognition phases. In practice, mismatched conditions are influenced by various lengths of spoken sentences, different types of recording devices, and the usage of different languages in training and recognition phases. All tests conducted in this research were performed in laboratory conditions using the specially designed framework for multimodal biometrics. The obtained results show consistency with the findings of recent research which proves that i-vectors and solutions based on probabilistic linear discriminant analysis (PLDA) continue to be the dominant speaker recognition approaches for text-independent tasks.

## 1. Introduction

In a world where Internet services have become the backbone of Internet usage, identity management systems are an important part of everyday life. Automatic speaker-recognition systems, as part of identity management in many e-commerce applications, in addition to general business interactions, have emerged as a pertinent means of identity verification [1]. Two important tasks of all identity management systems are to verify user identity verification and access control. The performance of the access control system directly depends on the certainty of establishing the identity of the user accessing the system. Generally, someone’s digital identity can be based on the premises of who they are, what they have, or what they know. A major weakness of identity management systems based on objects in the user’s possession, such as an ID card, is system vulnerability to theft or object loss. In cases where the identity management system is based on checking something only the user and the system know, there is a risk of forgetting the key information or breaking the shared code with brute force, social engineering, or similar identity hacking techniques. One of the ways for reducing the likelihood of identity theft is to utilize biometrics which provides an answer to the question “Who are you?” [2]. At the same time, due to its probabilistic nature, the usage of biometrics introduces uncertainty to the verification process. However, the reliability of the claimed identity could be enhanced by utilizing more than one biometric modality [3].

The choice of biometric modality depends primarily on the nature of the application, the access device, the conditions in which the application is used, the required decision reliability, and the modality characteristics. For example, in mobile and home banking, transactions should be carried out using a mobile network, or the Internet, so it is convenient to use the human voice as one of the factors for user verification [4]. The need for voice-based user verification is increasing as chatbot popularity is emerging. In this scenario, a user communicates via text, and/or voice, with an AI-powered program [5].

With the increased interest in the use of voice as a method for user identification, the aim of this paper was to research the impact that a change in working conditions in the process of voice recognition has on the performance of identity verification and access control systems.

The human voice contains both physical and behavioral biometric characteristics [6]. As with any biometric system, the speaker identification system has two basic modes: enrollment and recognition/verification. In enrollment mode, a working set of speakers’ speech data is acquired. Afterwards, some salient characteristics of the speech are extracted from the data, and a database of the speakers’ voice characteristics is generated. In the recognition operating mode, the biometric system checks for a feature set enrolled in the database that matches the input feature set. In the verification operating mode, the biometric system checks if the input feature set matches a particular set enrolled in the database [2].

Even though voice recognition is a practical method for identity verification, it faces various difficulties, such as a variation of speakers’ vocal tract characteristics, emotional states, traits of the microphones used, a noisy environment, and the quality of the communication channel between the time of enrollment and future recognition.

More robust models for speaker identification, non-matching enrollment, and recognition conditions have been published [7,8]. Based on these models, various solutions for speaker recognition have been implemented and intensively tested. The American National Institute of Standards and Technology (NIST) has organized a series of speaker recognition evaluations (NIST SRE) since 1996. Evaluations are conducted mainly in languages spoken by hundreds of millions of people around the world, so the question of the applicability of the evaluation results to other languages is still open. It is often necessary to adapt an application developed for one language area to another in a process known as localization.

However, localization of an e-commerce application with voice-based user identification may further downgrade the performance of the system. In a case where the user communicates with the application in a foreign language, it is necessary to examine how this affects the performance of the speaker recognition system because localization of the application has not been carried out [9,10].

This paper presents an evaluation of the performance of four popular open-source speaker recognition solutions (SPEAR, MARF, ALIZE, and HTK) of the various enrollment and recognition conditions. Mismatched conditions are influenced by the varying lengths of spoken sentences, types of recording devices, and the differences between training and operating languages. All the tests were performed under laboratory conditions using the specially developed framework for multimodal biometrics [11]. The main premise of this research arises from the assumption that mismatched conditions in enrollment and recognition phases influence the performance of speaker recognition system. Although most of the current work addressing this problem focuses on evaluating the performance of different speaker recognition algorithms, this research focuses on variation in the enrollment and recognition phases of the speaker recognition process.

The next chapter gives an overview of the state of the art in the area of speaker recognition. In chapter three, the most popular open-source solutions for speaker recognition are presented. The following chapter describes the setup of the experiment performed as part of this research, with the results presented and discussed in the next section. Finally, conclusions are given based on the discussion and results.

## 2. State of the Art

The speaker recognition process uses essential speech information to claim the speaker’s identity. The fundamental components of any speaker recognition system are the speaker models and speaker feature extraction algorithms. The speaker model faithfully represents a speaker and is created in the system based on the speech feature vectors. Feature vectors are derived from the input speech by applying one of the extraction algorithms.

In the enrollment mode, a speaker model is trained using the feature set derived from the utterance of the target speaker [12]. Different modelling techniques are available, and their usage depends on the type of speech used in the speaker recognition process. In that context, two main approaches to speaker modelling are used:

1. Text-dependent speaker recognition;
2. Text-independent speaker recognition.

For each of the speaker modelling approaches, the relevant literature suggests applying different modelling techniques. In text-independent speaker recognition, popular modelling techniques include:

- Gaussian mixture models (GMMs);
- Neural networks.

Using Gaussian mixture models is not a new technique, but it is very efficient when it comes to speaker recognition. GMMs are used in many applications that are involved in statistical data analysis, pattern recognition, computer vision, voice and image processing, and machine learning [13].

Neural networks are a new approach in speaker recognition, and they are starting to take precedence in the field. Several types of neural networks are used in the speaker recognition process. Richardson et al. concluded that deep neural networks (DNNs) are very effective in speaker recognition [14]. Villalba et al. described neural networks as the best performing act for speaker recognition [15]. In their paper, “State-of-the-art Speaker Recognition with Neural Network Embeddings in NIST SRE18 and Speakers In The Wild Evaluations”, they show that in some conditions, x-vectors’ detection error reduces by two i-vectors. An x-vector model is a deep neural network that generates one single vector or embedding per utterance, characterizing the speaker [16].

Neural networks speaker modelling approaches are widely implemented and used in commercial applications. IDVoice, a product of the ID R&D company, with headquarters in the United States, combines proprietary x-vector technology, deep neural networks (DNN), convolutional neural networks (CNN), and their own patented p-vector technology to achieve exceptional performance [17]. VoiSentry is a voice biometric product by United Kingdom based company called Aculab which uses a hybrid approach to leverage the best state-of-the-art artificial neural networks (ANNs) and analytical linguistic and signal processing technology, to achieve multilingual authentication in real time [18].

When it comes to text-dependent speaker recognition, the best practices are systems based on hidden Markov models. The traditional hidden Markov model (HMM)-based systems give DNNs comparable results in terms of speaker recognition accuracy. Unfortunately, DNNs require a large training sample [19].

It is important to describe the parameters which are known as *de facto* standards for evaluating the performance of a speaker recognition system. Two parameters widely used for the evaluation of biometric recognition systems are recognition accuracy and recognition speed. Accuracy can be quantitatively described by the following measures:

- False acceptance rate—FAR;
- False rejection rate—FRR;
- Equal error rate—EER.

The false acceptance rate (FAR) measures the likelihood that the biometric system, based on the calculated score and chosen threshold, inaccurately decides that the input feature set and the feature set selected from the database belong to the same person. The false rejection rate (FRR) measures the likelihood that the biometric system, based on the calculated score and chosen threshold, inaccurately decides that the input feature set and the feature set selected from the database do not belong to the same person. The equal error rate (EER) refers to a specific operating point of the biometric system where the FAR equals the FRR. It should be noted that the biometric system with the lowest EER value has the best decision-making performance [2].

## 3. Speaker Recognition Open-Source Solutions

In the reference literature, there are various open-source speaker recognition solutions. Each of them follows at least one speaker modelling approach and implements at least one speaker feature extraction algorithm. Some of the most popular open-source speaker recognition software packages and toolkits include SPEAR, MARF, ALIZE and HTK. Different approaches to speaker recognition problem-solving in those solutions allows evaluation of, not only the performance under the same operating conditions, but also the evaluation of algorithm sensitivity to the variations in parameter values that define those operating conditions.

SPEAR is an open-source solution and contains an extended set of speaker recognition tools. It is based on Bob Toolkit, which contains free signal processing and machine learning components. The toolkit supports several modelling techniques: Gaussian mixture models (GMMs), intra-session variability (ISV), common factor analysis, and total variability (i-vectors). It is developed in C++, but is also provided in the Python environment [20]. SPEAR also provides two feature extraction algorithms for extracting spectral and cepstral characteristics: mel-frequency cepstral coefficients (MFCCs) and linear frequency cepstral coefficients (LFCCs).

MARF (Modular Audio Recognition Framework) is an open-source platform that contains a collection of voice, sound, speech, text, and natural language processing algorithms written in Java. MARF includes several feature extraction algorithms such as fast Fourier transform (FFT), linear predictive coding (LPC), artificial neural network (ANN), as well as various distance classifiers [21]. In terms of speaker recognition, this framework provides support for text-independent speaker recognition.

The ALIZE open-source toolkit for speech recognition is based on Gaussian mixture modeling with a universal background model (GMM-UBM). It supports state-of-the-art techniques, such as: joint factor analysis (JFA), support vector machine (SVM), i-vector modeling, and probabilistic linear discriminant analysis (PLDA) [22]. This toolkit is designed to be able to handle high volumes of speech and language data.

HTK (HMM Toolkit) is a toolkit generally used to build and model hidden Markov models. In the HMM-based speech recognition system, each word is represented by speech vectors. It is assumed that a Markov model generates the sequence of observed speech vectors corresponding to each word. This toolkit consists of training and recognition processes. Specifically, it supports several sub-steps: data preparation, training, and recognition [23].

Considering the popularity of open-source solutions, as well as the different algorithms they implement for extracting speaker features, the authors of this research have selected them as perfect candidates to compare and evaluate the performance of speaker recognition systems in different environments with varying parameters. The setup of this experiment and its specifics are described in detail in the next chapter.

## 4. Setup of the Experiment

The main goal of the experiment was to examine how the mismatch between enrollment and recognition conditions influences the performance of the speaker recognition system. Thus, the experiment was performed in two phases:

- The enrollment phase;
- The recognition phase.

During the enrollment phase, the valid user’s speech data were collected for setting up the selected speaker recognition models. Subjects spoke the sentences following the provided text instructions, thus representing valid users of the system. The collected speech data were recorded in Serbian and English. Serbian was chosen because existing works lack research which include this language, whereas English was chosen as the language most used in similar speech and speaker recognition experiments.

In the recognition phase, training data were compared to new input voice data in order to evaluate the performance of the speaker recognition system.

There was a total of 40 subjects, of which 30 represented valid users and enrolled the training data. Training data were gathered from the subjects in laboratory conditions. The other 10 subjects were representing imposters and provided data during the recognition phase. All of the subjects fell under the age group ranging between 18 and 35 years. Out of 30 valid users, 16 were female and 14 were male.

During the data acquisition, both in the enrollment and recognition phases, the subjects were simultaneously recorded with three devices:

- An integrated microphone on a laptop;
- An external microphone on a desktop computer;
- A 3G mobile phone.

Detailed specification of the used recording devices is presented in Table 1.

Subjects were fluent in Serbian and English, and were recorded in both languages. Textual instructions in both Serbian and English were created for recording purposes.

Text prompts were separated by length and by context. There were three lengths:

1. Short (up to 1 s);
2. Medium (1 to 2 s);
3. Long (over 2 s).

Text statements were divided by context into three categories:

1. The first set of statements contained the voices of characters, numbers, and basic commands that simulated interaction with the operating system and the menu;
2. The second group of sentences utilized terms usually found in the banking vocabulary, for instance, “Do you offer cash loans indexed in Serbian Dinars?”;
3. The third group of sentences consisted of terms used in everyday life situations, such as the names of beverages, food, the time of day, common actions, etc.

All words and sentences were collected in both Serbian and English. By using the collected data of the valid users, each chosen speaker recognition model was trained separately in English and Serbian.

Testing and evaluation of the system was performed in the recognition phase by using valid users’ utterance, as well as 10 additional new users,’ which imitated the role of imposters.

## 5. Results and Discussion

Before presenting the results of our research, we will first give a brief overview of a possible way to evaluate the performance of a biometric system.

The efficiency of the biometric system is reflected in the accuracy and speed of decision making. The calculation of the similarity score between the two specified feature sets is the basis for deciding whether or not both data sets belong to the same entity. If the calculated score is above the chosen value, named as the threshold, we believe that both feature sets belong to the same entity. We say that both feature sets are a case of intra-class variation. Of course, great attention should also be paid to the speed at which the scores are made. Due to the many comparisons performed, slower methods should not be used in the identification mode.

To test the sensitivity of the open-source speaker recognition solution to language mismatch, both English and Serbian were used as different languages in the enrollment and recognition phases. There were a total of four scenarios using the enrollment and recognition steps:

1. English in both phases;
2. English in enrollment and Serbian in the recognition phase;
3. Serbian in enrollment and English in the recognition phase;
4. Serbian in both phases.

For each scenario, measurements were made for nine different working conditions, determined by the length of the spoken text (short, medium, and long) and the used recording device (integrated microphone, external microphone, and 3G mobile phone).

**Figure 1:**
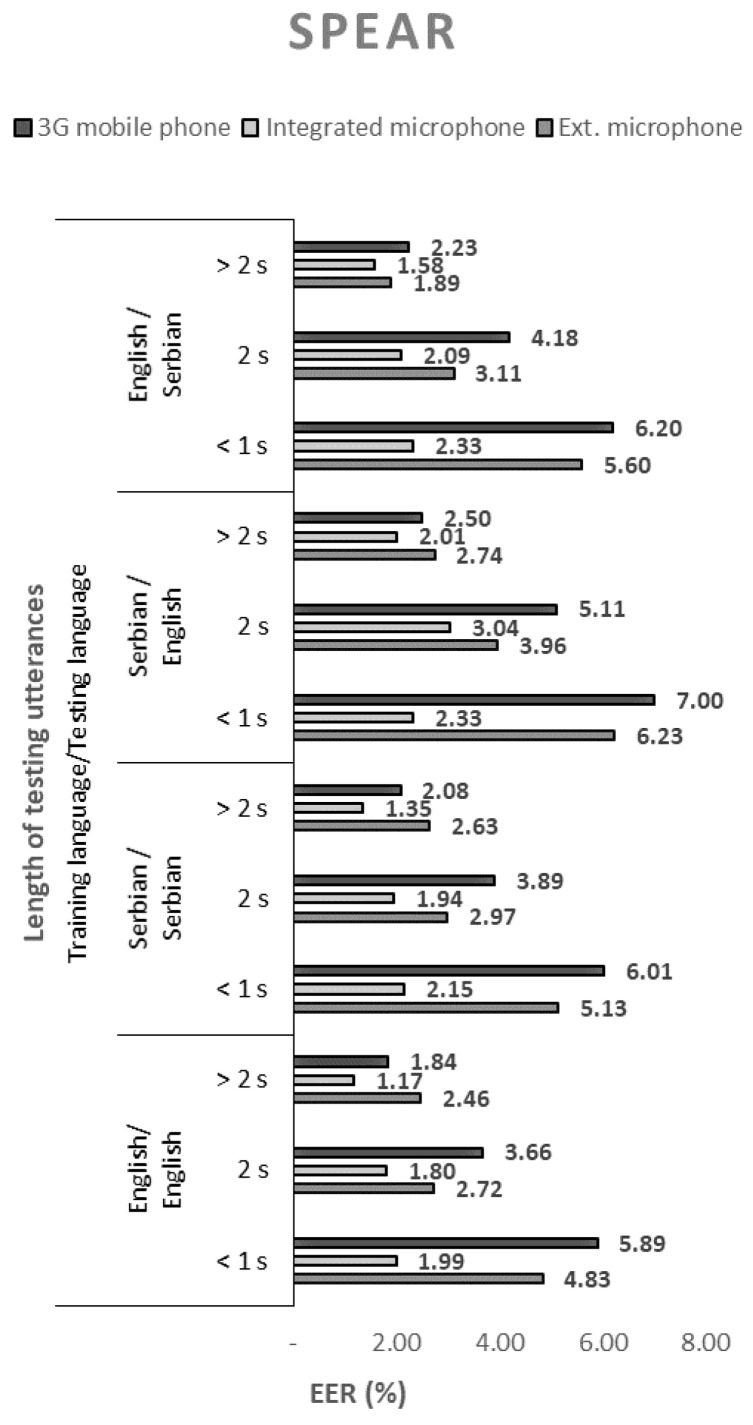
Equal error rates for the SPEAR speaker recognition model.

**Figure 2:**
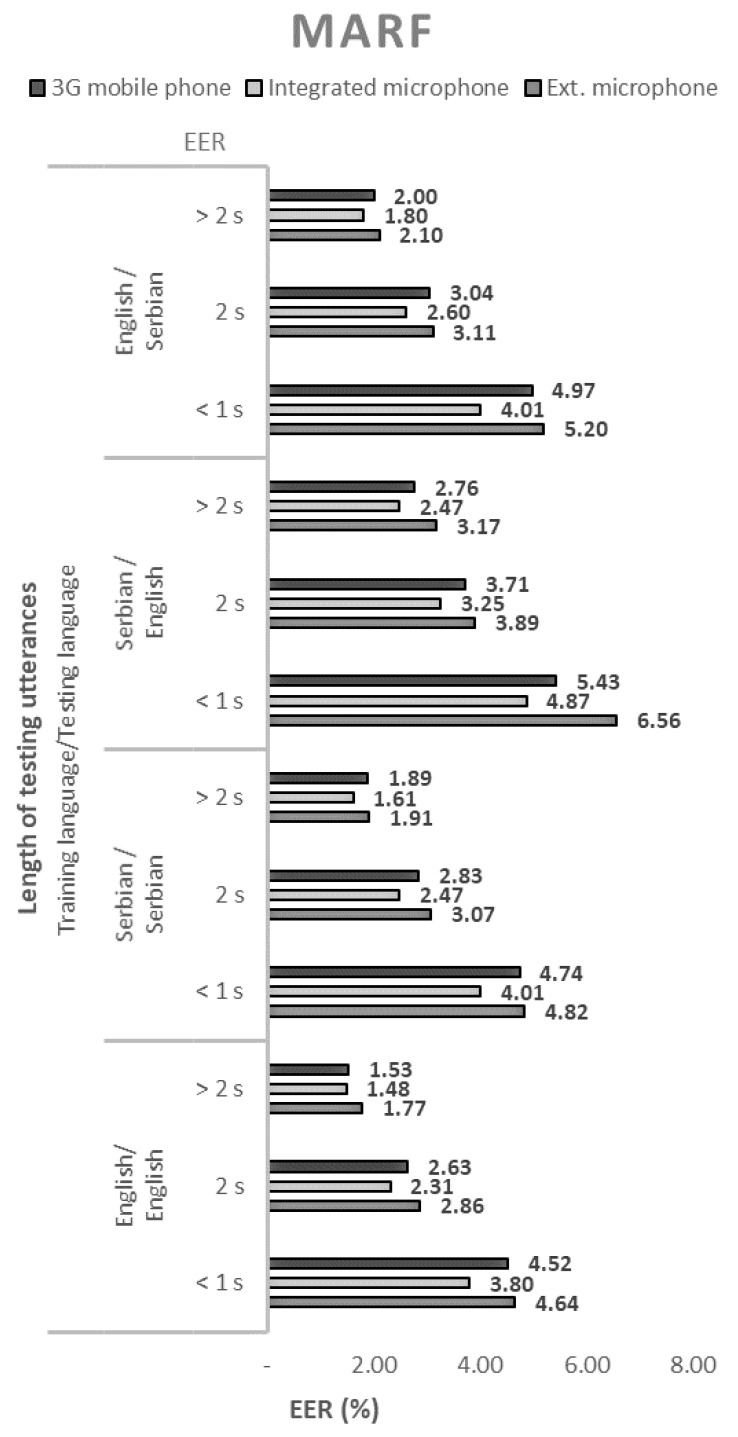
Equal error rates for the MARF speaker recognition model.

**Figure 3:**
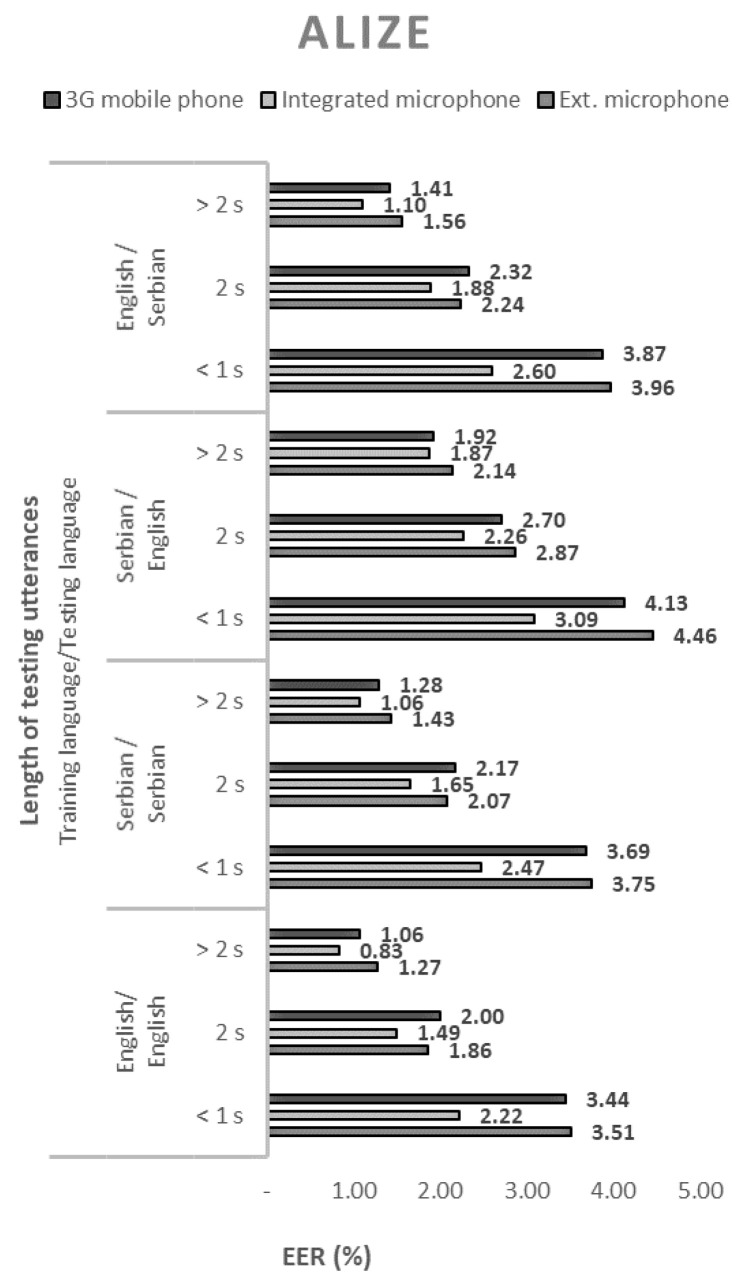
Equal error rates for the ALIZE speaker recognition model.

**Figure 4:**
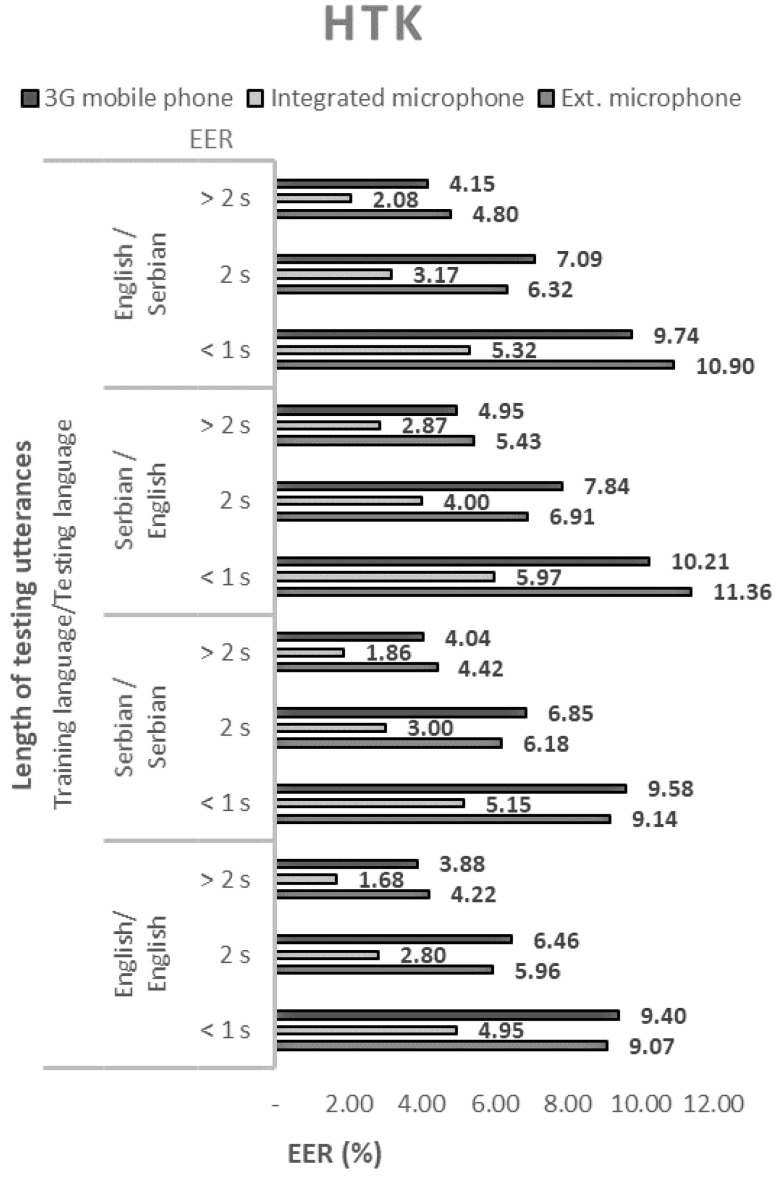
Equal error rates for the HTK speaker recognition model.

The performance of the 144 tested configurations with open-source solutions, measured by the equal error rate (EER), are shown in Figure 1, Figure 2, Figure 3 and Figure 4.

The best performances of the speaker verification system grouped by solution are shown in Table 2, Table 3, Table 4 and Table 5. Each table shows a measured EER percentage for one solution and each language combination.

When using SPEAR, it is notable that using Serbian language in enrollment and English in the testing phase results in a slightly higher EER percentage. Similar conclusions can be drawn for the case where MARF is used.

By analyzing all of the obtained results, the following conclusions can be drawn regarding the selected open-source solutions:

- The ALIZE open-source toolkit for speaker recognition showed the best performance in all language combinations. Speaker recognition software tool, SPEAR, came in second place, and MARF’s open-source platform came in third. The weakest speaker recognition scores were obtained using the HMM Toolkit, HTK;
- All four tested open-source solutions gave the best results with a combination of English as both a training and test language. Somewhat weaker results were obtained in cases where Serbian was used as a training and test language. In these cases, the EER increased from 9% for MARF to 28% for ALIZE.

It is interesting to note that, in the case of a mismatch between a training and test language, better results were measured for the combination of English as a training language and Serbian as a test language. The EER values for the configuration of Serbian as a training language and English as a test language increased from 27% for SPEAR to 70% for ALIZE, compared to a combination of English as a training language and Serbian as a test language.

In all cases, regardless of the open-source solution used, the language, and the voice recording device, the ERR values decrease monotonically with the length of the test sequence. Therefore, the best performance of the speaker recognition system is obtained for the longest test sequence. By extending the sentences from short records (up to 1 s) to long records (over 2 s), the EER decreased by an average of about three times.

The EER values shown in Figure 1, Figure 2, Figure 3 and Figure 4. show that the devices used for voice recording significantly affect the performance of the speaker recognition system. In the case of this experiment, under laboratory conditions, the best results were measured with a microphone integrated into the laptop; slightly worse with the use of a 3G mobile phone; and the worst with a cheap external microphone connected to a desktop computer. For example, the combination of ALIZE and an integrated microphone results in 1.5 times lower EER for all mismatch configurations using an external microphone.

## 6. Conclusions

This research aimed to determine how the performance of a system for identity verification and access control, based on speaker recognition techniques, is influenced by chosen open-source technologies, various lengths of spoken sentences, types of recording devices, and mismatch between a training and an operating/test language. This study examined the performances of four popular speaker recognition system solutions that implement different approaches and techniques to the problem under consideration. After analyzing the results of the speaker recognition system, obtained in 144 different tested configurations, the conclusion was that the most successful open-source speaker recognition solution is ALIZE. The ALIZE open-source toolkit for speaker recognition is based on Gaussian mixture modeling with a universal background model (GMM-UBM). It supports techniques with i-vector modeling and probabilistic linear discriminant analysis (PLDA). The results obtained with the ALIZE and SPEAR packages are consistent with the findings of recent research that i-vectors and PLDA-based solutions continue to be the dominant speaker recognition approaches for text-independent tasks [24].

The fact that the results are significantly better when English was used both as a training and test language suggests that the solutions considered were optimized for the use of English. If the tested open-source software is used with another language, the optimal values of the configuration parameters in the software package must be carefully examined before it is used. This implies that the performance of speaker recognition methods varies by language.

The analysis of the results shows that the values of the EER parameter in the first approximation are inversely proportional to the length of the sentences. Additional research should indicate the optimum sentence length at which the minimum possible EER is approached.

Tested software solutions are very sensitive to the characteristics of the voice recorder, which in practice can significantly affect the performance of the speaker recognition or verification system. It is desirable to use a solution that, in preprocessing, minimizes the impact of variations in the characteristics of the speech recording device. Additionally, the results presented in this paper indicate potential problems that may occur when localizing a chatbot application requiring speaker verification.

In further research, it would be interesting to examine the performance of speaker recognition systems which use voice as one of the biometric modalities, in two-factor authentication (2FA) or multifactor authentication (MFA).

## Figures and Tables

**Table 1:** Recording device specifications.

Device	Configuration	Format
Desktop computer with external microphone	Computer model: Fujitsu, Intel^®^ Core^TM^ i5-2320 CPUMicrophone model: HYUNDAI CJCM30	WAV
3G mobile phone	Samsung S4 (GT-I9505)	WAV
Integrated microphone on a laptop	Model: HP ProBook 650 G1, Intel^®^ Core^TM^ i3-4000M CPU	WAV

**Table 2:** The best SPEAR performance, as measured by EER, compared to the languages used.

SPEAR			
EER %		Testing Languages	
		Serbian	English
Training languages	Serbian	1.35	2.01
	English	1.58	1.17

**Table 3:** The best MARF performance, as measured by EER, compared to the languages used.

MARF			
EER %		Testing Languages	
		Serbian	English
Training languages	Serbian	1.61	2.47
	English	1.80	1.48

**Table 4:** The best ALIZE performance, as measured by EER, compared to the languages used.

ALIZE			
EER %		Testing Languages	
		Serbian	English
Training languages	Serbian	1.06	1.87
	English	1.10	0.83

**Table 5:** The best HTK performance, as measured by EER, compared to the languages used.

HTK			
EER %		Testing Languages	
		Serbian	English
Training languages	Serbian	1.86	2.87
	English	2.08	1.68

## Data Availability

Data available on request due to restrictions eg privacy or ethical. The data presented in this study is only available in aggregate form and on request from the corresponding author. The data are not publicly available due to sensitive nature of biometric data.
